# Pathological Changes in the White Matter after Spinal Contusion Injury in the Rat

**DOI:** 10.1371/journal.pone.0043484

**Published:** 2012-08-29

**Authors:** C. Joakim Ek, Mark D. Habgood, Ross Dennis, Katarzyna M. Dziegielewska, Carina Mallard, Benjamin Wheaton, Norman R. Saunders

**Affiliations:** 1 Department of Pharmacology, University of Melbourne, Parkville, Victoria, Australia; 2 Department of Neuroscience and Physiology, Sahlgrenska Academy, University of Gothenburg, Gothenburg, Sweden; University of North Dakota, United States of America

## Abstract

It has been shown previously that after spinal cord injury, the loss of grey matter is relatively faster than loss of white matter suggesting interventions to save white matter tracts offer better therapeutic possibilities. Loss of white matter in and around the injury site is believed to be the main underlying cause for the subsequent loss of neurological functions. In this study we used a series of techniques, including estimations of the number of axons with pathology, immunohistochemistry and mapping of distribution of pathological axons, to better understand the temporal and spatial pathological events in white matter following contusion injury to the rat spinal cord. There was an initial rapid loss of axons with no detectable further loss beyond 1 week after injury. Immunoreactivity for CNPase indicated that changes to oligodendrocytes are rapid, extending to several millimetres away from injury site and preceding much of the axonal loss, giving early prediction of the final volume of white matter that survived. It seems that in juvenile rats the myelination of axons in white matter tracts continues for some time, which has an important bearing on interpretation of our, and previous, studies. The amount of myelin debris and axon pathology progressively decreased with time but could still be observed at 10 weeks after injury, especially at more distant rostral and caudal levels from the injury site. This study provides new methods to assess injuries to spinal cord and indicates that early interventions are needed for the successful sparing of white matter tracts following injury.

## Introduction

Injuries to the spinal cord, which are most prevalent in young people, often result in permanent life-long impairments of motor, sensory and autonomic nervous system functions. These can have serious negative impacts on the physical and emotional health of affected people as well as on their families. There are very few treatments available to improve the outcomes of spinal cord injuries. So far most research has been focused on trying to stimulate new axonal growth through the injury site in order to restore some degree of neurological function. However, it seems clear that several obstacles have to be overcome before such an approach could lead to any significant improvement of motor/sensory functions. These include the requirement for neuronal processes to traverse the site of the lesion including the glial scar before making functional connections on the other side of the injury and re-engaging intact peripheral nerves that have lost some function due to inactivity. The biological processes in such reconnection are poorly understood, largely because the number of axons growing beyond the site of injury is usually small, irrespective of the intervention used to provoke such growth. Therefore it may well be more feasible to aim to improve outcomes of spinal cord injury (SCI) by limiting the damaging effects of pathophysiological events, which occur in the aftermath of the insult. Our previous study has shown that the majority of grey matter loss in the spinal cord following a contusion injury occurs within hours of the injury and is mostly complete by 24 hours [1]. This may be too short a time window for effective intervention to be practicable in many clinical circumstances. In contrast, loss of white matter appears to extend over several days post injury [1]. This longer period provides an opportunity for treatments aimed at axon-sparing to be effective in retaining functional connections. However, a better basic knowledge of these pathological events is needed in order to develop and evaluate new treatment regimes. These needs are evident given that decades of SCI research have given numerous pre-clinical studies reporting various strategies to improve the outcome of spinal injuries, which have unfortunately not been translated into the clinical setting [2], [3].

Deficits in neurologic function below the level of a SCI are thought to be mostly due to the loss of white matter in and around the injury site [4]. However, most SCIs are not complete and there are large differences in the amount of spared cord tissue. White matter injuries result in loss of long ascending and/or descending tracts reducing communication between brain and peripheral organs and between different levels of the spinal cord. These deficits affect systemic motor and sensory functions, but also frequently cause loss of bowel and bladder control making a normal lifestyle difficult. An attractive option of effective SCI treatment is therefore to reduce the damage to white matter tracts in order to preserve connections that had survived the initial injury, but are subsequently lost due to secondary injury processes. The present study follows on from our earlier one in which we compared the general loss of grey matter and white matter following a contusion injury [1]. The new study was undertaken to better our understanding of pathological events occurring in the white matter following injury and to test new methods for assessing damage to spinal white matter.

## Methods

### Ethics statement

All animal experiments were conducted following NH&MRC guidelines and were approved by the University of Melbourne Animal Ethics Committee (Ethics#0703702).

### Surgery and spinal cord injury

A position and velocity controlled impactor device was used to generate defined spinal injuries (described in detail in [1]). It consists of a linear motor (LinMot) that is mounted on a manipulator arm of a stereotaxic frame and computer controlled via a servo unit. The experimental design was identical to that described previously [1]. Briefly, anaesthetised (3% isoflurane) young adult rats (8 weeks) were subjected to single contusion spinal injuries at thoracic (T) spinal level 10. An impactor tip of 2 mm diameter penetrated to 2 mm below the dura at a velocity of 1 m/s and with a dwell time of 100 ms. Animals were allowed to recover and administered analgesic (buprenorphine 0.06 mg/kg) daily for the first 4 days after injury. At different times after injury (between 2 hours to 10 weeks), animals were processed for histological examination using either of two fixation protocols. In one group, spinal cords from SCI and sham operated controls (laminectomised without contusion) were fixed with paraformaldehyde/Bouin's fixative and embedded in paraffin wax for light microscopical examination. In a second group of animals, spinal cords from SCI and naïve age-matched controls were fixed with a paraformaldehyde/gluteraldehyde mixture and embedded in resin for electron and light microscopic examination [1]. In the present study white matter in and around the injury site was analysed in detail, using staining of oligodendrocytes (CNPase) and myelin (Luxol Fast Blue) together with counts of axonal numbers, examination of axonal pathology and ultrastructural changes in white matter after injury.

### Immunocytochemistry

At different times after spinal cord injury (2-, 24-hours, 4 days, 4- or 10-weeks), the injured rats and age-matched sham controls (n = 4 per group) were terminally anaesthetised with isoflurane inhalation (>10%), perfuse-fixed with a 4% paraformaldehyde solution, a 10 mm spinal cord segments, centred on the injury side were dissected out, post-fixed in Bouin's fixative for 24 hours and then processed for histology as described previously [1]. Serial 5 µm transverse sections were cut and used for immunocytochemical analysis using antibodies for the oligodendrocyte marker CNPase (2′,3′-cyclic nucleotide 3′-phosphodiesterase; mouse monoclonal antibody diluted 1∶200, Sigma). Antibody dilutions were made in phosphate buffered saline, PBS/Tween20 (pH7.4). Every 10th slide was stained for CNPase by incubation in primary antibody overnight at 4°C followed by incubation in rabbit anti-mouse (DAKO in 1∶200 dilutions in PBS/Tween20) and mouse PAP (DAKO, 1∶200 dilution in PBS/Tween20). The reaction product was developed with DAB+ staining kit (DAKO), dehydrated and mounted with Ultramount (Fronine). Control slides incubated without the primary antibodies were always blank.

### Luxol Fast Blue staining

Myelin in tissue sections was stained with Luxol Fast Blue (LFB). Sections from the centre of the injury were selected along with sections from 2 and 5 mm rostral and caudal to the injury site. The sections were de-waxed and cleared in 100% and 95% ethanol before staining in a solution of Luxol Fast Blue MBSN (0.1% w/v, Aldrich), 10% acetic acid and ethanol (95%) overnight at 60°C. Sections were rinsed in 95% ethanol and differentiated in 0.05% lithium chloride solution followed by 70% ethanol. Differentiation was stopped by washing in distilled water until unmyelinated tissue appeared white.

### Light and electron microscopic examination of plastic sections

Injured animals at 24 hours, 1- 4- and 10-weeks after injury (n = 3–4 per group), along with age-matched uninjured controls, were killed with inhalation of isoflurane (>10%). A 25 mm long segment of spinal cord centred on the injury site was removed from the animals, divided in the middle in order to ensure a cord surface from the middle of the injury, and cut at every 1 mm using a vibrating microtome. Tissue blocks were processed as for electron microscopic examination in order to preserve the myelin (details in [1]). Semi-thin sections (0.5 µm) at every millimetre from 10 mm rostral to 10 mm caudal of the injury were collected, stained with methylene blue and examined in the light microscope. Ultrathin sections of selected areas of interest were cut with a Reichart-Jung microtome, contrasted with uranyl acetate/lead citrate and examined under a Phillips CM10 electron microscope.

### Estimation of the number of axons and axonal pathology

The number of myelinated axons in the dorsal column (DC) and in the ventrolateral tracts (VLT) was estimated in the 0.5 µm thick plastic sections using a motorised Stereology system with Stereoinvestigator 7.0 software as previously described [1]. Only myelinated axon profiles with diameters above 0.5 µm and below 10 µm were included in the counts. Many axonal profiles in the spinal injured animals showed pathology with swollen axons and detached myelin sheaths. These were estimated in separate probing runs (see below). The upper limit of 10 µm diameter excludes axons with severe pathology (no myelinated axons in uninjured age-matched control animals exceed 10 µm diameter) and the lower limit of 0.5 µm is the smallest diameter that axons can be individually identified with light microscopy.

In an initial run the number of axons was determined in all tissue blocks (10 mm rostral to 10 mm caudal) from animals at 24 hours and at 10 weeks in order to determine the overall spatial pattern of white matter damage. Subsequently for intervening time points, tissue sections at the centre of injury site, 2 mm rostral/caudal and 9 mm rostral/caudal were used for stereological probing runs. These sites were chosen to determine the number of myelinated axons at the centre of injury site, towards the outer edges of the primary injury zone and from well outside the primary injury.

The number of axons that showed clear signs of pathology was also estimated at 1-, 4- and 10-weeks following spinal injury. All axons with a diameter larger than 10 µm were included in counts as well as those where ≥25% of the axon was detached from its myelin sheath. In both the dorsal columns and ventrolateral tracts the number of these axons was estimated using 30 counting frames at 1200 times magnification. In addition, the number of empty/collapsed myelin sheaths (no axon visible) was estimated in the same probing run. This appears to be a later stage of the pathological process (see Fig. S1) and may have important implications since myelin is thought to inhibit the capacity for new fibre growth. The electron microscope was initially used in order to confirm that we could correctly differentiate these abnormal myelin sheaths in the light microscope from other tissue structures with similar appearances. Using the stereological software the spatial distribution of axons exhibiting pathology was also mapped.

### Image analysis

Images of sections stained for CNPase were captured with a DP-70 Olympus digital camera attached to an Olympus BX-50 microscope. The area of positive staining was measured using ImagePro plus. For CNPase the whole cord was outlined and automatic dark object recognition used to measure CNPase positive areas of the sections.

### Statistical analyses

All data are presented as mean ± standard error of the mean (SEM) unless otherwise specified. Data were analysed using analysis of variance (ANOVA) and individual differences were determined using Bonferroni's post-test.

## Results

### General morphology of white matter after spinal cord contusion injury

A general description, especially of grey matter, of the morphology of the spinal cord after contusion injury has been published previously [1]. This section refers specifically to white matter using the same experimental material and starts with a general description of morphological changes in spinal columns 24 hours to 10 weeks post injury followed by a more detailed analysis of axonal pathology.

#### Dorsal column

**Figure 1:**
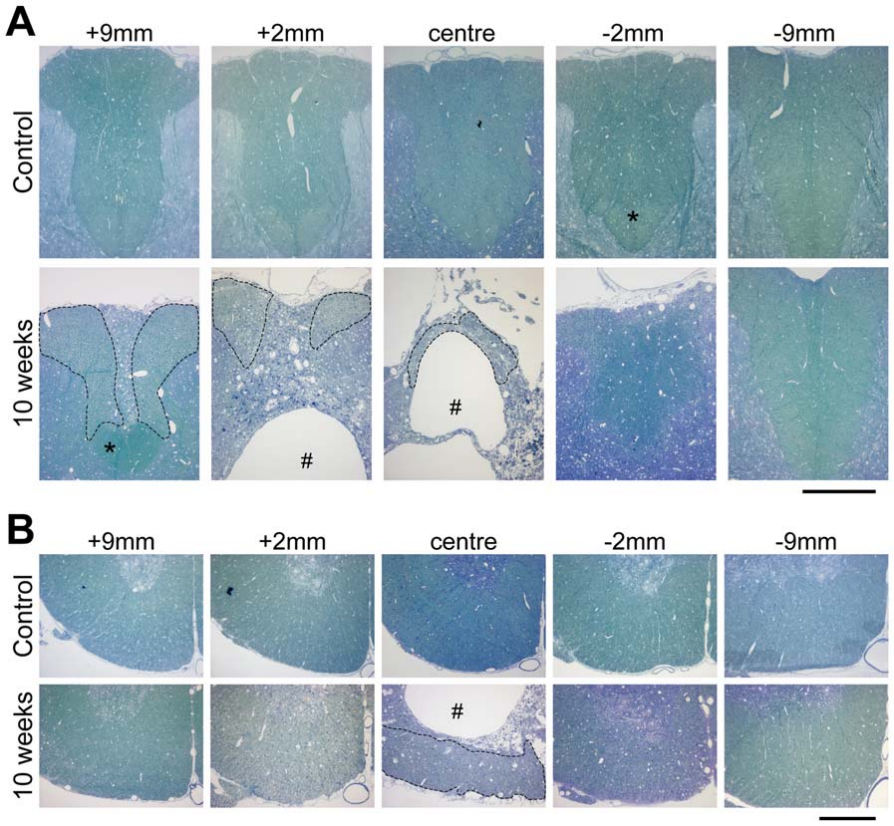
Plastic sections (0.5 µm) stained with methylene blue of aged matched control and at 10 weeks after injury rat spinal cords. (A) dorsal column, (B) ventrolateral tracts at different distances from injury centre. The #-symbol marks the cyst that develops after the injury. In the dorsal column at 10 weeks after injury only a small area is left in which axons are visible (area marked with dotted line); this area becomes progressively larger rostrally. Caudal to the injury the dorsal column is little affected; however, the corticospinal tract that runs at the base of the dorsal column (marked with an asterix) is not visible caudal to the injury but in contrast appears normal rostrally. Scale bars are 300 µm.

At 24 hours after injury most of the dorsal column (DC) was filled with cell debris in the middle of injury site (not illustrated). Some areas of the most dorsolateral parts of the column appeared less damaged. Damage extended to about 3 mm on either side of the centre of the injury; whereas further away, the gross morphology of the DC appeared normal. At 1-week post injury there was much more cell and myelin debris in the centre of the injury and some macrophages were present in the medial parts of the DC; part of this area had been cleared of tissue and a central cyst cavity had started to form (not illustrated). Further rostral, the central part of the column appeared filled with degenerated axons, but lateral to this, the white matter appeared normal and moving further rostral to the injury the area of normal white matter increased. Caudal to the primary injury zone the DC appeared largely unaffected. The appearance of the white matter tracts at 4 weeks was similar to that illustrated for 10 weeks (Fig. 1); only a small part of the DC remained at the injury centre located at the outer rim of cord immediately underneath the pial surface. A large cyst cavity was visible in the middle of the cord containing many macrophages (identified by electron microscopy). Rostral to the primary injury zone, the central core of the DC had degenerated to a similar extent to that seen in animals at 1-week post-injury. The corticospinal tract, which in rats lies at the base of the DC, appeared intact rostral to the primary injury site, but was absent caudal to the injury site. At 10 weeks post-injury (Fig. 1), the DC appeared similar to that observed at 4 weeks, however there were few macrophages in the central cystic cavity.

#### Ventrolateral tracts

At 24 hours, in the injury centre, damage was visible in the ventrolateral tracts (VLT) and was more severe in the more central parts of the cord. Areas closer to the pial surface appear mostly unaffected. Longitudinally, damage extended to about 3 mm either side of the injury centre and further away the gross morphology appeared similar to age-matched controls (not illustrated). At 1 week in the injury centre a large amount of cell debris was present in the more central areas of the VLT. Extensive damage was visible in some peripheral areas although some areas appeared unaffected. Damage to the cord was visible at all spinal levels studied, however at distal levels to the injury, the peripheral areas of the VLT appeared more affected than the more central areas; this is in contrast to the primary injury zone where the central areas are more affected. At 4 weeks, only an outer rim of the VLT remained at the injury centre and a similar appearance was seen at 10 weeks (illustrated in Fig. 1). No further significant changes were observed between 4 and 10 weeks.

### CNPase immunoreactivity

**Figure 2:**
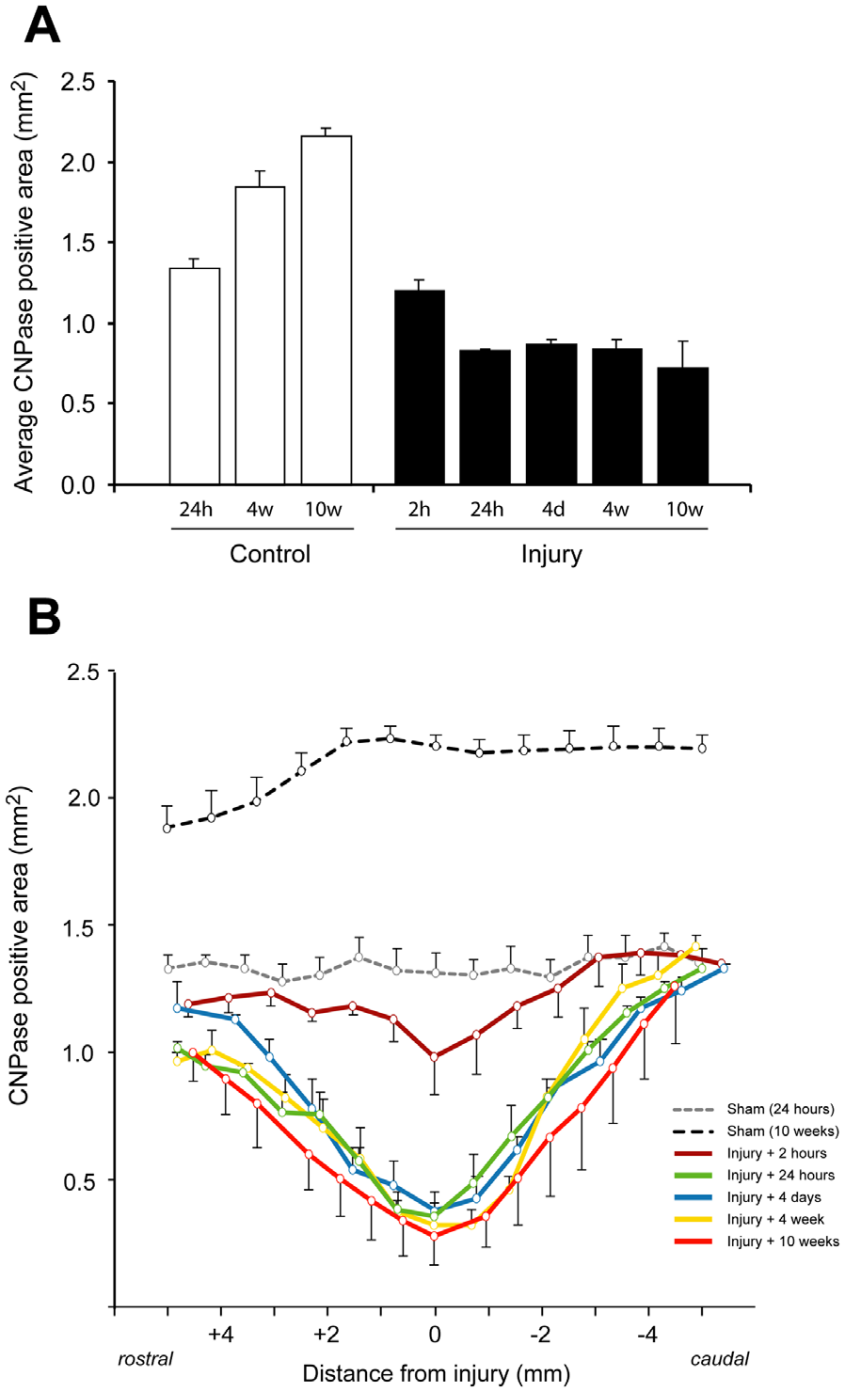
Area of CNPase immunoreactivity in tissue sections of spinal cords in control and injured animals. **A**) Bar graphs represent average positive area (mm^2^) in tissue sections (12–14 per animal) between 5 mm rostral to 5 mm caudal of injury centre (±SEM, n = 3–4 animals per group). Open bars are age matched controls and closed bars are injury groups. In controls the CNPase area progressively increased with age of rats from around 1.4 mm^2^ (24 hours sham controls) to 2.2 mm^2^ (10 weeks after injury). At 2 hours the area was slightly lower than 24 hours sham controls but was significantly lower at all later stages in the injury group cords, however, no significantly difference was found between 24 hours and 10 weeks after the injury. 2 h – 2 hours, 24 h – 24 hours, 4 d – 4 days, 4 w - 4 weeks, 10 w – 10 weeks. **B**) CNPase area at different distances from centre of injury. At 2 hours after the injury the area is similar to controls except around middle of the injury (approximately1 mm on either side) where it is lower. In all other injury groups the CNPase area is well below controls in the centre of injury and up to 4–5 mm caudal and appears somewhat lower even up to 5 mm rostral to injury. Data are mean ± SEM.

The area of positive CNPase immunoreactivity, indicative of oligodendrocytes, in spinal cord sections from 5 mm rostral to 5 mm caudal of the injury site was determined between 2 hours and 10 weeks after injury and compared with sham operated control animals. The data are presented in Figure 2A as the average area over the whole segment of the cord, calculated from serial sections, in each control and spinal injury group. This shows that as the uninjured animals grew and matured, there was a progressive increase in the immunopositive area from 1.34±0.06 mm^2^ at 24 hours, to 1.85±0.09 mm^2^ at 4 weeks and to 2.17±0.05 mm^2^ at 10 weeks (open bars). In animals at 2 hours after spinal cord injury, there was a small decrease in the immunopositive area (1.22±0.09 mm^2^), but the difference was not statistically significant. At all later times after injury, significantly smaller CNPase positive areas were observed compared to age-matched sham operated controls (p<0.05). The values obtained were similar between 24 hours and 4 weeks (0.85–0.86 mm^2^) and smaller at 10 weeks (0.73±0.16 mm^2^) after injury, however, this difference was not statistically significant.

In Figure 2B the same data for CNPase positive areas are presented at different levels of the cord centered on the injury. At the middle of the injury site (0 mm) there was a small, but significant, decrease in the immunopositive area as early as 2 hours after injury (0.98±0.14 mm^2^ compared to 1.32±0.14 mm^2^ in age-matched controls, P<0.05). However, at >2 mm rostral and caudal, the immunopositive area was similar to the age-matched control values. At 24 hours after injury, there was a marked decrease in the immunopositive area at all levels between 2 mm rostral and 2 mm caudal with the middle of the injury site reduced to approximately 27% of the age-matched control value (0.36±0.06 mm^2^ compared to 1.32±0.14 mm^2^, P<0.01). There were no further significant reductions in the CNPase positive area at the middle of the injury between 24 hours and 10 weeks. The values from 24 hours to 10 weeks were all significantly lower than at 2 hours after injury (p<0.05). At 4–5 mm caudal to the middle of the injury site, the area of CNPase positive staining was similar to sham operated controls in all of the injury groups, whereas at 4–5 mm rostral the area was always somewhat smaller than in controls in all injury groups.

**Figure 3:**
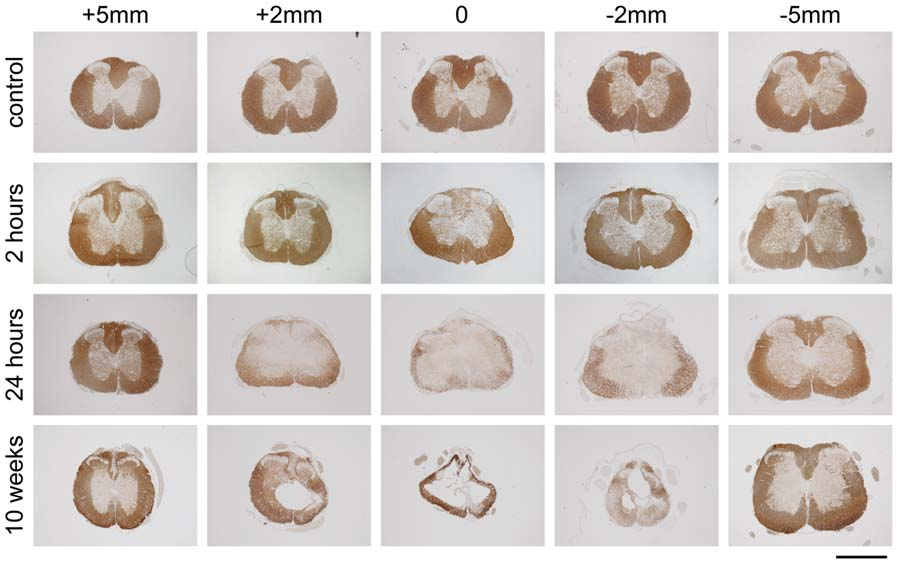
Illustrations of CNPase immunoreactivity in 24-hour sham controls, 2-, 24- and 10-weeks after injury. In control animals immunoreactivity is mostly confined to white matter of spinal cord. At 2 hours after injury immunoreactivity is lost in the dorsal column and the apex of ventromedial parts of white matter in the centre of injury. At 24 hours after injury, nearly all immunoreactivity is confined to an outer rim of the cord in the centre of injury. Immunoreactivity extends further towards the middle of the cord away from the injury site and at 5 mm rostral/caudal the pattern is similar to control cords. At 10 weeks after the injury only a small rim of tissue is left in the centre of the injury and nearly all this tissue is CNPase immunoreactive. At 5 mm caudal, the pattern of staining is similar to controls and likewise at 5 mm rostral expect for little immunoreactivity in the most medial parts of the dorsal column. Scale bar is 1 mm.

The typical staining pattern of CNPase after injury is illustrated in Figure 3. All sham operated controls (24 hours to 10 weeks) showed a similar staining pattern with dark staining of all the white matter and some lighter staining in the grey matter (only 24 hour shams illustrated). At 2 hours after injury, the CNPase immunoreactivity was visibly reduced in the dorsal column and in some animals was also somewhat reduced at the apex of the more medial parts of ventral white matter, however, this was only detected between 2 mm above and 2 mm below the injury site (see also Figure 2B). At 24 hours after injury, CNPase immunoreactivity was almost completely absent in the dorsal column up to about 3–4 mm rostral and caudal from the injury site. At the middle of injury site (0 mm), only the outer most layers of the ventrolateral white matter showed a thin area of immunopositive staining, this area became progressively larger with increasing distance from the centre of the injury site. At 10 weeks after injury, only a thin rim of tissue remained surrounding a cystic cavity that had formed in the middle of the injury site and which extended both rostrally and caudally. Almost all this remaining tissue was immunopositive for CNPase. In all of the injured animals, the CNPase staining pattern at 5 mm from the centre of the injury site was similar to that in sham operated controls (Fig. 3).

### Luxol Fast Blue

**Figure 4:**
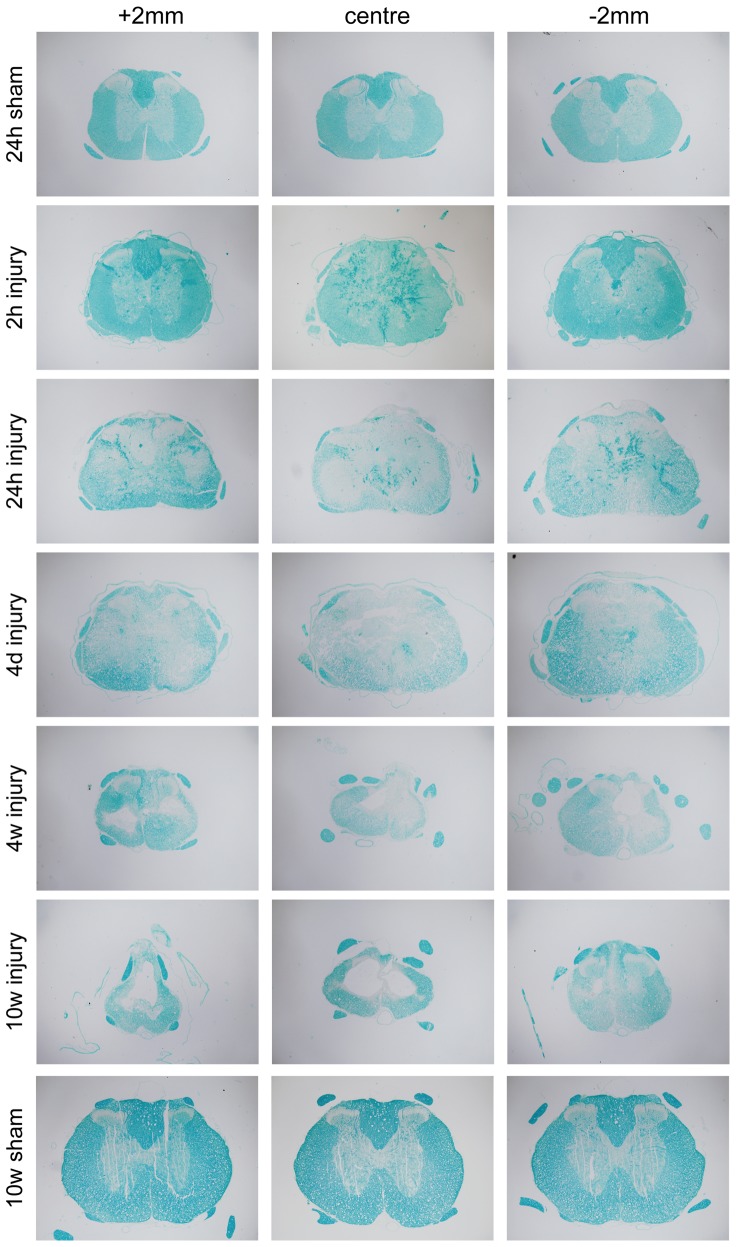
Illustrations of Luxol Fast Blue (LFB) staining of paraffin sections at different times after injury along with 24 hour and 10 week sham controls. At 2 hours after injury LFB staining appears similar to sham controls animals in white matter although blood clots also stain positive for LFB; these can be seen up to 4 days after the injury making it more difficult to interpret the results from these times. At 24 hours the LFB positive area is greatly reduced and in the centre of injury there is faint staining on the outer edges of the ventral white matter (similar to CNPase staining, see Fig. 5). At 4 and 10-weeks after the injury, most of the tissue that is left is LFB positive Note also the larger LFB positive area in 10-week shams compared to 24-hour shams.

In sham operated control animals, Luxol Fast Blue (LFB) staining of myelin in the spinal cords was as expected, mostly restricted to the white matter (Fig. 4). Unfortunately blood clots also stained with LFB; which made it difficult to interpret the results obtained from most animals at 2- 24-hours after injury and also in some of the cords at 4 days; this made automated measurements of LFB positive area impossible. Nevertheless, from direct microscopical observations it appeared that LFB staining was little affected at 2 hours. At 24 hours and 4 days, LFB staining was much reduced with almost no staining visible in dorsal column at the centre of the injury and more granular staining visible in the ventrolateral white matter, which was more prominent at the outer edges of cord. At 4 weeks after injury, most of the remaining tissue was positive for LFB and at 10 weeks almost all remaining tissue (a thick rim around the periphery of the cord) was LFB positive in (Fig. 4).

### Numbers of myelinated axons, their area and density

Stereological based microscopy was used to estimate the number of myelinated axons in both the dorsal column and ventrolateral tracts at different times and different levels of the spinal cord relative to the centre of the injury site. Axon areas and axon densities were also determined.

**Figure 5:**
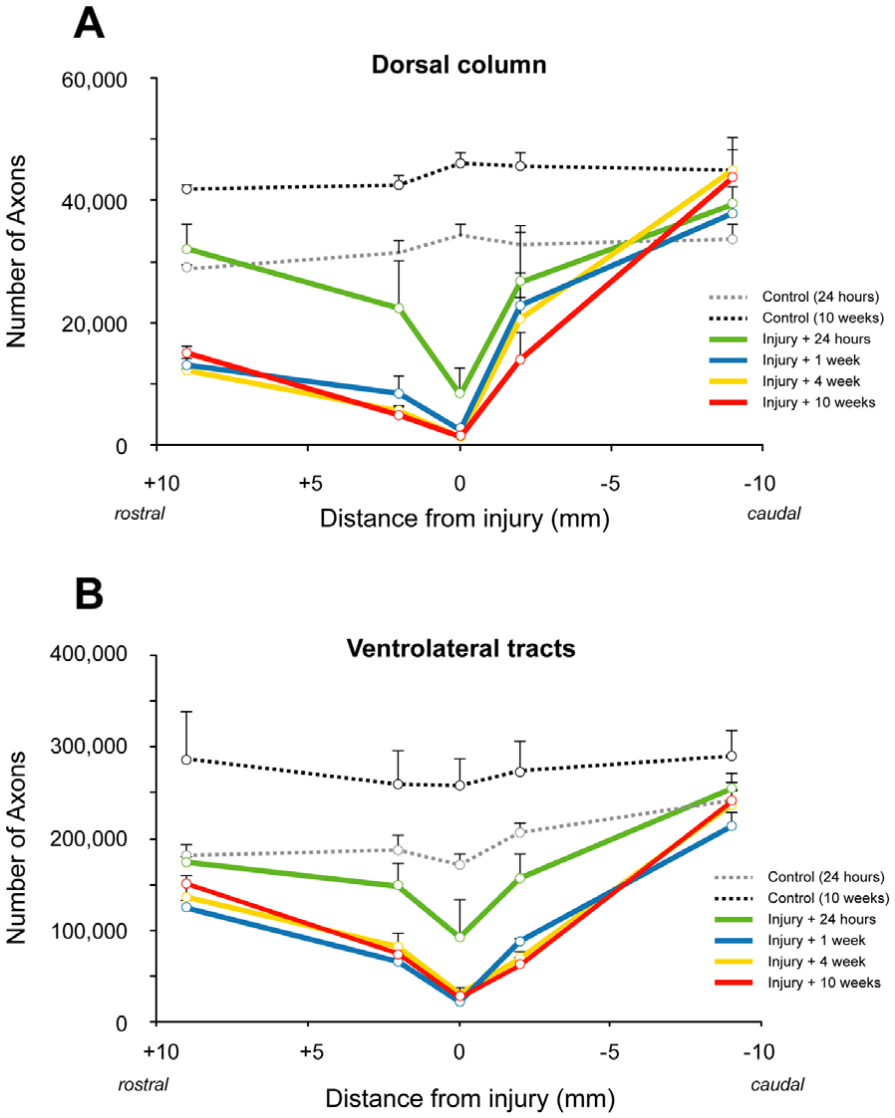
Number of myelinated axons in the dorsal column (A) and ventrolateral tracts (B) in control and spinal injured rats. **A**) In the middle of the injury site at 24 hours after the injury, the number of axons is reduced to 23% of controls (0 week) and further reduced to 4–6% at later times after injury. The number of axons was similar in animals at 1–10 weeks after the injury at all distances from injury and was much lower than controls even at 9 mm rostral to injury (43–52% of 0 week controls) whereas at 9 mm caudal numbers were similar to controls. Note that in 10-week controls the number of axons is about 38% (or 12,000 axons) more than in 0-week controls in the dorsal column. **B**) In the middle of the injury site at 24 hours after the injury, the number of axons is reduced to 54% of controls (0 week) and further reduced to 13–17% at later times after injury. The number of axons was similar in animals at 1–10 weeks after the injury at all distances from injury and was lower than controls at 9 mm rostral (71–83% of 0 week controls) whereas at 9 mm caudal it was similar to controls. Note that in 10 week controls the number of myelinated axons is about 40% (or 76,000 axons) more than in 0 week controls in ventrolateral tracts.

The results for axon numbers are presented in Figure 5. The average number of myelinated axons (−9 to +9 mm) in the dorsal column was 32,200±2,200 (mean±SD) in uninjured control animals of the same age at which the contusion injuries were made and increased to 44,200±1,900 (mean±SD) in animals that were 10 weeks older (Fig. 5A). This represents an average increase of 38% (±2.3%) throughout the whole length of the cord segment during the 10-week period. Following spinal injury, the number of myelinated axons was significantly reduced. By 24 hours after injury, the number of axons in the dorsal column at the middle of the injury site had reduced to around 24% of controls (8,100±3,700; p<0.01). By 1 week, the number of remaining axons in the dorsal column at the centre of the injury site was around 13% of controls (2,400±700; P<0.01). There were no further significant reductions in axon numbers in the dorsal column at the injury centre between 1 and 10 weeks after injury. At 9 mm caudal to the centre of the injury site, the number of dorsal column axons was not significantly different from age-matched controls at all times after injury. At 9 mm rostral to injury site, the number of axons was similar to age-matched controls at 24 hours, but significantly lower (p<0.05) at all other times up to 10 weeks.

In the ventrolateral white matter tracts, the average number of myelinated axons (between −9 to +9 mm) was 197,600±27,800 (mean±SD) in uninjured control animals of the same age at which the contusion injuries were made and increased to 273,900±15,100 (mean±SD) in the oldest control group of animals (age-matched for 10 weeks post injury, Fig. 5B). This represents an average increase of 40±6.7% in the number of ventrolateral white matter myelinated axons along the length of the cord segment. In the middle of the injury site, the number of axons at 24 hours after the injury was reduced to less than 50% of control animals (91,900±37,600, P<0.05). By 1 week after injury, the number of axons in the ventrolateral white matter at the middle of the injury site had further reduced to around 13% of controls (21,700, P<0.01). There were no further significant reductions in the number of ventrolateral white matter axons between 1 week and 10 weeks after injury. At 24 hours after injury, the numbers of ventrolateral white matter axons at 9 mm rostral and 9 mm caudal to the centre of the injury site were similar to those in age-matched control animals. At later times after injury (>24 hours), axon numbers remained similar to age-matched controls at 9 mm caudal to the injury centre (174,100 at 1 week, 240,800 at 10 weeks), but were significantly reduced to just over half the age-matched control animals (105,400 at 1 week, 151,000 at 10 weeks, P<0.01) at 9 mm rostral to the injury centre.

Results for the cross-sectional area of the cord occupied by myelinated axons are presented in Figure S2. The area of the dorsal column (DC) increased from 0.341 mm^2^ (±0.04 SD) to 0.567 mm^2^ (±0.04 SD) in the age-matched controls, an increase in area of around 67% over the 10-week period (Fig. S2A). The cross-sectional area of the ventrolateral tracts (VLT) increased from 1.873 mm^2^ (±0.23 SD) to 2.936 mm^2^ (±0.18 SD) in the age-matched controls, an increase of around 57% over the 10-week period (Fig. S2B).

At 24 hours post-injury, the areas of the DC and VLT at the centre of the injury site and at 9 mm rostral and 9 mm caudal were all similar to age-matched controls. By 1 week post-injury, there was a significant reduction in the areas of the DC and VLT at the centre of the injury site, but not at 2 mm or 9 mm rostral and caudal. At 4 weeks post-injury, the area of the VLT at all points along the spinal segment was not significantly different from at 24 hours post-injury. In contrast, the area of the DC was similar to that at 24 hours post-injury only at the injury centre and at 9 mm rostral. At 2 mm either side of the injury centre, the area of the DC was significantly lower and at 9 mm caudal was significantly higher, although still lower than in age-matched controls. At 10 weeks post-injury, the area of the DC was similar to that seen at 4 weeks at all points along the spinal segment except for 9 mm caudal where it was increased. The area of the VLT was similar to that seen at 4 weeks at all points along the spinal segment except for 2 mm caudal where it was further decreased.

**Figure 6:**
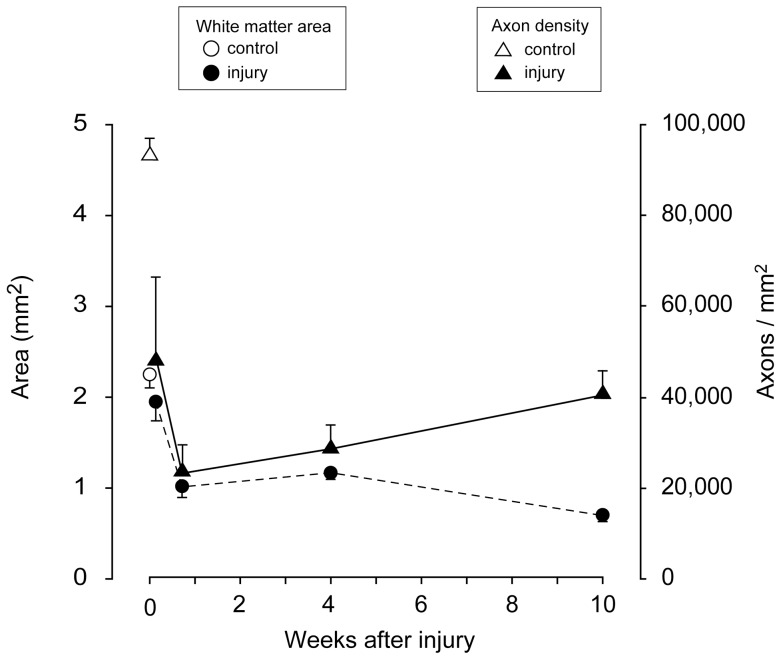
Area of myelinated fibres (closed circles, left y-axis) and axon density (closed triangles, right y-axis) at different times after the injury (x-axis). Open symbols represent control animals.

The temporal patterns of changes in the area and density of axons in the whole spinal cord cross section at the centre of the injury site are shown in Figure 6. Between 24 hours and 1 week, there was a rapid decrease in both axon density and the area of white matter staining. Between 1 and 10 weeks post injury, white matter area continued to decrease, albeit at a slower rate, whilst the density of axons gradually increased.

### Axonal pathology

The degree of axonal pathology was first assessed under the electron microscope. Four main stages were identified and these are illustrated in Figure S1. In age-matched control spinal cords, the largest diameter myelinated axons found within the white matter were around 6–7 µm. After spinal cord injury, many axons swell (stage 1) and the axoplasm became denser with organelles clustering in the centre of the axon. The myelin sheath detached from the axon (stage 2) and the axoplasm became less dense compared to stage 1. The axon then disintegrated leaving a microcyst inside an empty myelin sheath (stage 3). The myelin sheath sometimes appeared morphologically intact, but wrappings were often less densely packed than in control tissue. Typically intact axons were often found next to necrotic axons. In the last stage (stage 4), the large empty myelin sheaths became thinner with fewer wrappings before disappearing completely.

### Quantitation of Pathology

**Figure 7:**
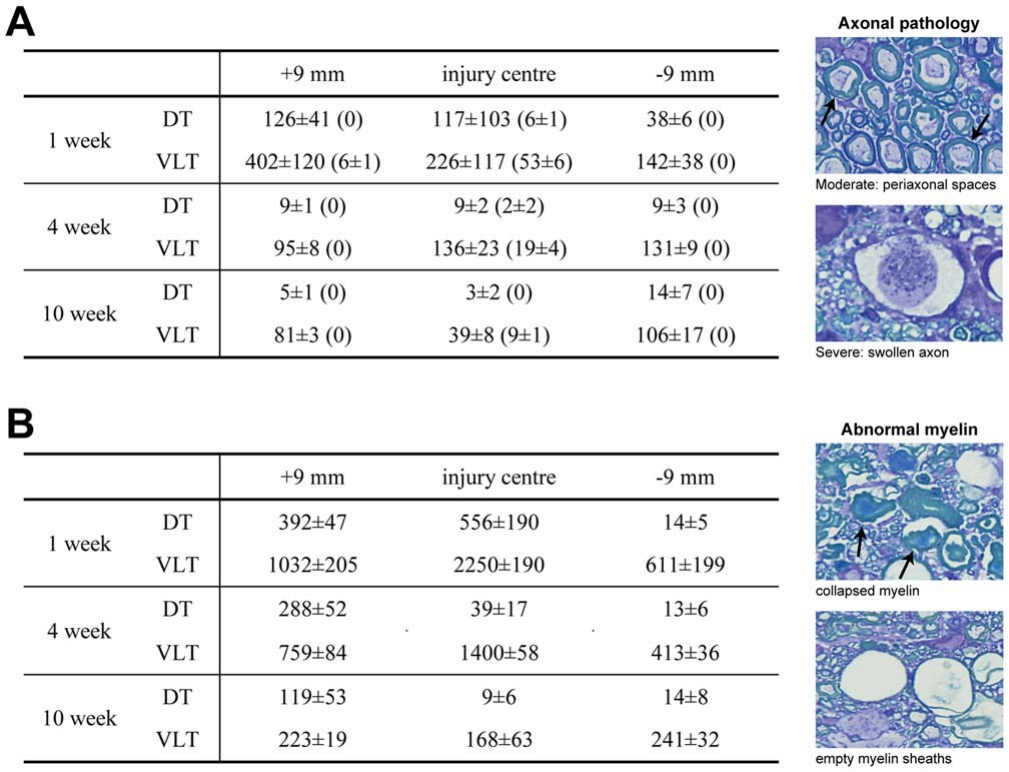
**A**) Number of axons with moderate pathology at 1-, 4- and 10-weeks after SCI in dorsal tracts (DT) and ventrolateral tracts (VLT). Numbers in brackets are axons exhibiting severe pathology. Note: These numbers should not be regarded as absolute numbers but rather as indicative of relative differences (see Methods). Examples of pathology are shown on right. Data are mean ± SEM (n = 3–4 at each time). **B**) Number of empty/collapsed myelin sheaths (examples shown on right) at 1-, 4- and 10-weeks after SCI in dorsal tracts (DT) and ventrolateral tracts (VLT). Data are mean ± SEM (n = 3–4 at each time).

In order to obtain a quantitative estimate of the numbers of axons with moderate (25–75% of myelin sheath detached) and severe pathology (defined as >75% of myelin sheath detached and/or severely swollen axon), semi-thin plastic sections were examined at 1-, 4-, and 10-weeks after injury at high magnification under the light microscope (Figure 7A). These numbers are likely to be an underestimation of the true number of axons with pathology since some pathological changes may not be detectable at the light microscope level. Nevertheless, these numbers are likely to reflect the general temporal pattern of changes in pathology in the injured cords. In sections from age-matched control cords, between zero and a maximum of four axons that showed some detachment of their myelin sheath were found, presumably reflecting a degree of artefact due to the fixation or subsequent processing.

#### Dorsal column

At 1 week after injury, the number of axons exhibiting pathology was similar at the injury centre and at 9 mm rostral (117±103 vs 126±41), but was significantly less at 9 mm caudal (38±6). At 4 and 10 weeks after injury, the numbers were very low at all levels studied (between 3 and 14). Axons with severe pathology were only ever found at the centre of the injury site at 1 and 4 weeks post-injury (6±1 and 2±2, respectively). No axons with severe pathology were observed at 10 weeks post-injury (Figure 7A, numbers in brackets).

#### Ventrolateral tracts

At 1 week post-injury, the number of axons with pathology was highest at 9 mm rostral (402±120), lower at the centre of the injury (226±117) and least at 9 mm caudal (142±38). The numbers of axons with severe pathology were highest in the middle of the injury site (53±6), with only a few present at 9 mm rostral (6±1) and none at 9 mm caudal. At 4 weeks the numbers were similar at all levels in the cord (around 100), but lower than at 1 week. Axons with severe pathology were only ever present in the centre of the injury site (19±4). At 10 weeks the numbers of axons were lowest in the centre of injury site (39±6), but higher at 9 mm rostral and 9 mm caudal (81±3 and 106±17, respectively). Axons with severe pathology were only ever observed in the centre of the injury site were present (9±1).

### Number of abnormal myelin sheaths

The number of empty or collapsed myelin sheaths was also estimated in semi-thin plastic sections at 1-, 4- and 10-weeks after injury (Figure 7B) in order to obtain another measure of the temporal pathological processes of the axon and its myelin.

#### Dorsal column

In the dorsal column the number of abnormal myelin sheaths was highest in the centre of the injury site (556±190), somewhat lower at 9 mm rostral (392±47) and only a few were observed at 9 mm caudal (14±5). At 4 and 10 weeks post-injury, the number was highest at 9 mm rostral (288±52 and 119±53 respectively) and much lower in the middle of injury and at 9 mm caudal.

#### Ventrolateral tracts

At 1 week after injury the numbers were highest in the middle of injury (2250±190), lower at 9 mm rostral (1032±205) and lowest 9 mm caudal (611±199). This pattern was similar at 4 weeks although at all levels of the cord the numbers were lower than at 1 week. At 10 weeks numbers were much lower than at earlier times and similar at all levels of the cords (around 200).

### Mapping of axonal pathology

**Figure 8:**
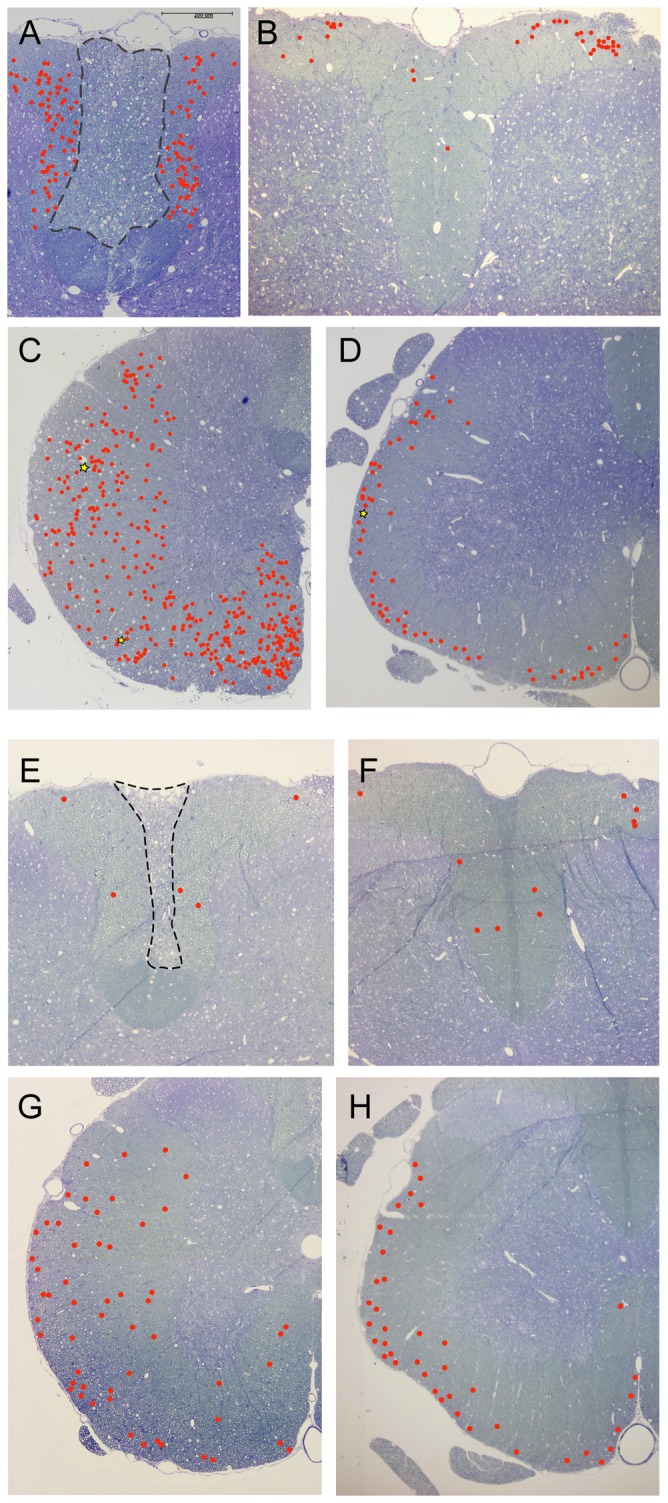
Illustrations of the general distribution of mapped axonal pathology at 1 week (A–D) and 10 weeks (E–H) after injury. Axons with moderate pathology are shown with red circles and axons with severe pathology with yellow stars. For illustrations of pathology see Figure 7. **A**) Axons in the dorsal column, 9 mm rostral of injury. Note that the pathology is uniformly spread throughout the white matter, outside the central core that is filled with degenerated axons (area inside dashed line). **B**) Axons in the dorsal column, 9 mm caudal of injury. Axon pathology is visible almost exclusively in the dorsolateral parts of the column where the axons enter from the spinal roots. **C**) Axons in the ventrolateral tracts, 9 mm rostral of injury. Axons pathology is uniformly spread within the white matter with some concentration to the medial parts. **D**) Axons in the ventrolateral tracts, 9 mm caudal of injury. Axon pathology is mostly restricted to the outer layers of the white matter close to the pial rim. Note that the numbers are much less than rostral to the injury. **E**) Axons in the dorsal column, 9 mm rostral of injury. A few axons are found outside the central core that is filled with degenerated axons (area inside dashed line). **F**) Axons in the dorsal column, 9 mm caudal of injury. A few axons are found throughout the column. **G**) Axons in the ventrolateral tracts, 9 mm rostral of injury. Axons pathology is rather uniform within the white matter. **H**) Axons in the ventrolateral tracts, 9 mm caudal of injury. Axon pathology is concentrated towards the pial rim in white matter and numbers are similar to caudal to injury (G).

In order to determine the spatial distribution of moderate and severe axonal pathology (see Figure 7 for example of pathology), the locations of these were mapped in cross sections at the centre and 9 mm caudal and rostral of the injury site at 1 and 10 weeks after injury. Representative sections are shown in Figure 8. At 1 week, rostral to primary injury site there was a central core of degenerated axons in the dorsal column (Fig. 8A). Axonal pathology was found on either side of this, uniformly distributed throughout the remaining dorsal column. Caudal to the primary injury site, axonal pathology was almost exclusively found only at the most dorsolateral parts of the column (Fig. 8B). In the ventrolateral tracts, axonal pathology was found throughout most of the tracts rostral to the injury site, but was more abundant towards the medial parts (Fig. 8C). In contrast, fewer axons with pathology were found caudal to the primary injury and these were concentrated towards the pial surface of the white matter (Fig. 8D). At 10 weeks after injury the distribution patterns of axonal pathology were similar to that at one week after injury both caudal and rostral to the injury site, but the overall numbers were much lower (Fig. 8E–H).

## Discussion

We have used a range of complementary techniques: immunocytochemistry, electron microscopy, estimations of axonal numbers, and axonal mapping at different times and at different levels of the cord, to detail the pathological events that occur in the white matter after a contusion injury to the rat spinal cord. The injury resulted in a rapid loss of axons that was mostly over by one week after the injury. Oligodendrocytes were affected very soon after the injury and detectable changes to oligodendrocytes preceded much of the loss of myelinated axons. Although most axon loss occurred within the first week, tissue reorganisation, as well as ongoing pathology was detectable up to 10 weeks after the injury, indicating that these events are long-lasting. Mapping of axonal pathology revealed distinct and contrasting patterns below and above the injury site. In general the pathological events occurred much faster in the middle of the injury site than at locations further away. These results add to our understanding of the pathological events following spinal injury and help to provide background information in the assessment of injury studies as well as new methods to assess outcomes of spinal injuries and possible therapies.

### General white matter pathology

There have been numerous studies of both the macroscopic and the fine structure of pathology of spinal injury in rodents as well as primates [5], [6], [7], [8]. In general, we could confirm many of the pathological events that these studies have shown. At 10 weeks after SCI, the complete loss of grey matter at the centre of the injury is still apparent and only very small portions of dorsal tracts and a rim of white matter in the VLT are still preserved (Fig. 1). A large cyst also developed over time within the centre of the cord. The cyst extended to about 2–3 mm away from injury site and a progressively larger rim of white matter was present in the VLT. Rostral to the injury in the dorsal column, there was a central core of tissue with a complete loss of axons and on either lateral side of this there was near to normal appearing white matter with myelinated axons. This area became larger more rostral to the injury site presumably because of axons that are entering the DC from higher levels and were little affected by the primary injury. In the DC caudal to injury, there was a complete loss of the corticospinal tract whereas other parts of DC appeared almost intact at some distance from injury site. The acute axonal pathology included a phase of dense organelle rich axoplasm often with a swollen axon, detachment of myelin sheath, complete degeneration of the axon along with unravelling of myelin sheath and disappearance of myelin altogether (Fig. S1). These abnormalities of the axoplasm preceding aberrations of the myelin are in agreement with previous studies [8].

### Oligodendrocyte pathology after SCI

The saltatory nerve signals in the CNS rely on myelination by oligodendroglia. We used CNPase immunoreactivity and LFB staining on tissue sections to detect changes to oligodendrocytes and myelin surrounding axons after SCI (Fig. 2, 3 and 4). Both staining methods consistently delineated white matter in the spinal cord of control animals. However, LFB also stained red blood cells present due to bleeding in the first few days following the injury, making quantification of the staining impossible. At later times, LFB differentiated white and grey matter well. CNPase positive areas were quantitated in the rostrocaudal axis from 2 hours to 10 weeks after the injury along with age-matched controls. In controls the area increased 61% between sham surgery and 10 weeks later, probably reflecting the normal increase in white matter during this period (discussed below). At 2 hours after injury there was some loss of CNPase immunoreactivity in DC extending 1–2 mm away from the injury site (Fig. 2 and 3) but otherwise staining appeared normal. At 24 hours there was a dramatic reduction of CNPase positive area extending to about 4–5 mm caudal of injury (Fig. 2). As seen in Fig. 2 both the total area of CNPase immunoreactivity and the area in the rostrocaudal axis did not change from 24 hours to 10 weeks after the injury. These data show that oligodendroglia are affected very early after the injury even at some distance from the primary site. A previous *in vitro* study of spinal cords excised from injured rats showed that the spread of changes to Ca^2+^ dynamics in oligodendrocytes can spread many millimeters from the injury site within hours [9]. Our data also suggest that the part of white matter that is finally going to be spared can be delineated very well with CNPase as early as 24 hours after the injury. CNPase may therefore be a useful way to assess the outcome of any therapeutic interventions in experimental animals. Although LFB was not quantified (see above), the early loss of LFB after the injury confirms that the pathophysiology of oligodendrocytes and their myelin starts very soon by the injury. Interestingly, at the most distal ends (both rostral and caudal of injury) that were analysed, CNPase positive area was never larger than in the 24-hour sham controls. This suggests that the normal myelination processes that are observed in control animals are greatly disturbed, even at some distance from the injury centre (Fig. 2B).

### Loss of myelinated fibres after SCI

In general it is believed that axon counts provide a superior quantitation of residual white matter than simpler methods such as area measurements [10]. Our findings strongly support this. We have used an un-biased stereology based method to estimate myelinated axon numbers at different spinal levels and times after injury. Previous studies of axon counts have been based on the original line-sampling method of Blight [11]. To our knowledge, we are first to use a stereology-based method to do this kind of analysis. Although it does require some initial testing to establish counting parameters, once these are done, the technique is relatively fast, thus enabling accurate resolution of axonal loss in different tracts. Total axon counts obtained here appear to be similar to those estimated previously for the rat spinal cord using the line-sampling method [10]. This technique could also be used to separately estimate numbers of differently sized axons as well as further discriminate between changes in different spinal tracts. However it should be noted that injuries to the spinal cord often make it very difficult to accurately delineate tracts due to physical distortions of the cord. In addition, unmyelinated fibres and myelinated axons of very small size are excluded.

Axons in the dorsal column mainly originate from dorsal root ganglion cells (except the corticospinal tract at the base of the dorsal column which was excluded from our analyses). Axon numbers in control animals increased over the 10-week period on average by about 38% in the dorsal column of the spinal cord sections studied. This may be due to a combination of an increase in the size of small already myelinated fibres (so that they are included in the counting) or new fibres being myelinated. Our data showed that after injury, there was a rapid loss of axons in the dorsal column at the centre of the injury site such that by 24 hours only 24% remained (Fig. 5). From 1 week and later there seemed to be no further loss of dorsal column axons. The degeneration of the distal part of DC fibres is clear given the asymmetrical loss of fibres above, but not below the injury site in all of the injured animals from 1 week and later. The number of fibres increased in the segment rostral to injury and this is presumably the result of axons entering the cord above the lesion site that were not damaged by the primary injury. Similar to the changes at the injury site, axon numbers were very similar at times between 1 and 10 weeks indicating that the degeneration of the distal part of these fibres is over by 1 week. In the caudal part of the DC, the numbers of axons suggest some progressive loss of fibres close to the injury whereas myelination/remyelination can occur at some distance from the injury. For DC data we initially estimated axon numbers at mm intervals and this showed that at about 4 mm rostral to injury, the axon counts for the 4 and 10 week groups were all above the 24-hour control group and progressively increased with rostral distance from injury site (not illustrated). This indicates that at least in this tract, at more distal levels rostrally, the normal myelinating processes are not much affected and continue after injury.

Fibres in ventrolateral tracts are mixed sensory and motor fibres and originate both below and above the injury site. There was a similar relative increase in the number of myelinated fibres in these tracts as in the DC in control animals over the 10 weeks of the study (40% increase in VLT vs 38% in DC). The overall relative loss of fibres was, however, less in VLT compared to DC. This can be expected since the impact injury was directly above the DC. In the middle of the injury site about 54% of fibres remained at 24 hours and this was further reduced to 13% at 1 week after injury of aged matched controls. Similar to the DC there was no indication of further loss of fibres beyond one week.

The total area of white matter in plastic sections was also measured (Fig. S2). The total white matter area significantly increased with age in control cords; there was a 67% increase in the DC and 57% increase in the VLT over the 10 weeks, corresponding very well with the 62% increase for total CNPase positive area (see above) during the same time. It is also evident that swelling of the cord is prominent in the DC up to 1 week after the injuries. In Figure 6, the area of white matter and axon density has been plotted for the middle of the injury site at different times after the injury. This shows that these lines are quite parallel from 24 hours up to 1 week after the injury indicating an initial correlation between axon numbers and white matter area. However, between 4 and 10 weeks post-injury the area of white matter continued to decrease whilst the density of axons contained within it increased. Thus at later times after injury simple measurements of spared white matter area do not provide an accurate estimate of the number of surviving axons. The increase in the density of axons suggests that the residual white matter at the injury centre is being compressed and that the fluid filled cystic cavity may have transformed into a pressurised syrinx. In this and most other experimental models of traumatic spinal cord injury, access to the cord is obtained via a dorsal laminectomy and there is subsequent scarring of the dura mater in this region. It is known from human studies that fibrous scarring of the meninges around the spinal cord is associated with the development of a pressure filled syrinx in the centre of the cord, which, as it expands, compresses surrounding tissue against the surrounding vertebral column [12]. Syrinx formation is also known to occur in about 28% of human SCI cases [13].

### Axonal pathology

We have estimated both the number of axons with visible pathology and also the number of empty myelin sheaths (axons completely degenerated) to obtain a quantitative measure of white matter pathology after the injury. To define axonal pathology we have used similar morphological events to [8] who focused on the acute pathology of axons in ventromedial parts after SCI. There are a number of interesting findings from our analysis. Firstly, the overall pathology was progressively reduced over time. Although pathology occurred at all levels of the cord, the reduction was more prominent in the middle of the injury than at levels distant from the lesion. The data for the DC showed an asymmetrical distribution with more pathology occurring rostral to the injury and almost no pathology caudal. This is of course expected since it is the segment of these fibres distal to the cell bodies in the dorsal root ganglia that degenerate. One week after the injury, the number of empty myelin sheaths was much higher than numbers of axons showing pathology; this is probably a reflection of the speed of axon degeneration after injury. This is also evident in the estimations of axonal numbers (see above). At 10 weeks after the injury, however, this is reversed and the amount of axonal pathology was less in the middle of the injury site than at distal levels. This reversal is likely to be due to the high rate of tissue clearance in the middle of the injury. A large number of macrophages were seen to invade the tissue, particularly abundant at one week after the injury, and these macrophages were often found to contain engulfed myelin debris and other parts of degenerated axons. Similar findings have been described before [5]. At distal levels to injury, no macrophages were seen to infiltrate the tissue, however these are not easily identified without specific cell markers unless present in high numbers. It therefore seems likely that the large, progressive decrease in the number of pathological axons/myelin over time that is evident in injury centre is a consequence of macrophages removing many of damaged axons as well as myelin debris. This removal of myelin is thought to be an important part of the healing and regeneration process of the spinal cord since myelin contains compounds that have been found to be inhibiting new fibre growth [14], [15], [16]. Thus the less efficient clearance of myelin debris outside the injury site may therefore be an important factor in the poor growth of new axons in this zone. Stimulation of macrophages to clear more myelin debris outside the centre of the injury could have a positive outcome for new fibre growth. Lotan et al [17] administered cytokines after optic nerve crush to increase the inflammatory response and promote macrophage recruitment. This led to increased neuronal nerve adhesion, thought to play an important role in nerve growth. Hirschberg et al [18] administered the anti-inflammatory agent dexamethasone after an optic nerve crush injury resulting in reduced growth of regenerating fibres but more sparing of tissue. Macrophages exist in different phenotypes, with activated macrophages divided into the M1-type, thought to be proinflammatory and the M2-type, thought to promote tissue repair and axon growth (reviewed by [19]). After SCI in mice it has been shown that there are both an M1 and M2 phenotype early after the injury (1–3 days) but later (>7 days), the M1 macrophages predominate (the type that is supposed to be less permissive for CNS repair [20]). Macrophages as well as other aspects of the inflammatory response therefore appear to be able to both have positive and negative effects on the outcome of injury to the CNS and we need to better understand this ambiguity for optimal therapeutic interventions.

A fundamental question is the time-frame and final outcome for the pathology of individual axons. For instance we do not know if the axons that show pathology at ten weeks have had that pathology from the time of the injury or if it was due to a late secondary injury process. It is also clear from our data that although the evidence of pathology subsides with time, some is still detectable up to 10 weeks after the injury. This ongoing pathology with time is in agreement with many previous studies, which have shown that demyelinating and apoptotic processes can occur for some time after SCI (reviewed by [21]). One long-term SCI study by Totoiu and Keirsted [22] in rats found a second wave of demyelination occurring several months after spinal injury. However, in our model of spinal injury in the rat, we could find little evidence that demyelination in the weeks after the injury has a significant effect on the total number of axons in and around the injury site although this does not rule out that it does not occur. Our results also suggest that a significant amount of myelination still takes place in young adult rats as part of normal development. Rats used in our study were aged 8 weeks at the start, weighing 170–200 g, increased to 350–450 g in spinal injured rats and to 450–600 g in un-injured or sham controls at 18 weeks (latest age after injury). The majority of spinal injury studies in rats use animals within the 200–300 g range [8], [22], [23], [24], [25], [26], with age often not specified. Therefore, the remyelination that has been shown after the injury in several of these studies may at least be partially explained by the normal myelination processes that occur in juvenile animals.

All axons that showed any type of pathology were mapped at 1 and 10 weeks after SCI (Fig. 7) at the most distal rostral/caudal level from the injury site. Sections closer to the injury site often showed great distortion of the cord and mapping was therefore not conducted. There was a clear pattern of pathology in the tissue sections. At the caudal end of the DC almost all the pathology was found at the most dorsolateral parts. This is where the DRG fibres enter the DC from via the spinal nerves. The axons with pathology are presumably fibres that come from parts of spinal nerves that actually originate from DRG at higher levels of the cord and their centrally projecting axons may have been damaged in the primary injury. This would mean that at 10 weeks there is practically no pathology in the fibres that come from levels more distal to the section. In the VLT the pathology was found quite uniformly throughout the white matter although some concentration was found at the most ventromedial parts. This is presumably because the medial region was most affected by the contusion injury, which is directly above the area and more lateral parts were less impacted. In contrast, in the caudal end of the VLT the pathology was concentrated in the outer rim of the white matter close to the pia.

### Demyelination and remyelination

There are numerous reports of demyelination and remyelination following SCI. The details vary, perhaps depending upon lesion used, time after injury and selection of areas reported on. There seems to be general agreement that the process of demyelination is slower and more protracted than following peripheral nerve injuries [27]. The process appears to involve either break-up of the myelin sheath and gradual engulfment by invading macrophages [28] and/or gradual thinning of the sheath to the point where some large unmyelinated axons could be observed [7], [23], [29]. Their very size makes the conclusion that these are derived from previously large myelinated axons seem reasonable. Less plausible are the claims by some authors that clusters of small unmyelinated axons were derived from previously myelinated axons [7], [22], [28]. In these reports, injured spinal cords show clusters of small axons of sizes corresponding to that of unmyelinated axons; it seems unlikely that groups of myelinated axons would simultaneously lose their myelin sheaths and shrink to the size normally associated with unmyelinated axons, leaving no trace of myelin debris. It appears that remyelination of demyelinated axons at chronic stages of injury may involve both Schwann cells, invading from dorsal root ganglia close to the site of injury [30] or immature oligodendroglia [31], [32], [33]. The extent to which demyelinated axons remyelinate either as part of “normal” post injury changes or in response to local application of potentially myelinating cells such as Schwann cells, olfactory ensheathing cells or progenitor oligodendroglia is important for devising and implementing repair strategies aimed at remyelination [29]. However, in devising such strategies it will be important to have clear evidence of what constitutes demyelinated axons and their successful remyelination. Horner and colleagues have questioned the notion that there is chronic widespread demyelination in the injured cord [34], [35]. In their studies injured and intact axons were differentiated after SCI and they found no evidence of demyelination of intact axons but that this only occurred in injured axons. They also found substantive endogenous remyelination of injured axons albeit this results in shortened internodes and thinner myelin to normal spinal tracts. Altogether the authors contested the idea that there is a large target population of demyelinated axons to manipulate for therapeutic improvements.

### Conclusions

We found rapid loss of axon numbers after SCI in rats with little evidence of further loss beyond one week after the injury although tissue remodelling continued for much longer. Changes to oligodendrocytes could be detected even earlier and CNPase in cords predicted very well the longer-term survival of white matter tissue. We also found evidence for continuing myelination in juvenile rats, which does not seem to have been considered in previous rat studies. These processes appeared to be arrested closer to the injury site but continued at distal levels. We found progressive decrease in pathology with time but it was still detectable up to 10 weeks after the injury. Pathological changes to axons were faster in the middle of the injury and also the clearing of myelin debris, which is likely to be due to the recruitment of macrophages to this area. Altogether it appears that there is a need for early intervention to have a significant outcome for sparing of white matter following injury, however, the exact timing for an effective intervention may of course be dependent on the injury type (i.e. contusion/compression injury) and model in which it is studied.

## Supporting Information

Figure S1 **Area of myelinated axons in the dorsal column (A) and ventrolateral tracts (B) in control and spinal injured rats.** In both dorsal column (DC) and ventrolateral tract (VLT) there is a trend for the area to be progressively smaller with time in the middle of the injury. At 9 mm rostral to the injury the area is similar to 0 week controls in all injury groups whereas at 9 mm caudal in the later injury groups (4 and 10 weeks) the area is closer to 10-week controls. Note that in 10-week controls the area is 67% more than in 0-week controls in the DC and 57% more in the VLT.
(TIF)

Figure S2 **Electron micrographs of axons at different pathological stages, illustrating what appears to be a common pathological process of necrotic axons.** In the control spinal cord the largest axons found within white matter are around 6–7 µm. After injury, many axons swell (stage 1) and the axoplasm becomes denser with organelles clustering in the centre of the axon. These swollen axons detach from the myelin sheath (stage 2) and the axoplasm becomes less dense compared to stage 1. The axon disintegrates and a microcyst is left in the space where the axons were situated (stage 3). It is interesting to note that morphologically intact axons are often found next to necrotic axons. The myelin sheath sometimes appears morphologically intact but wrappings are often less densely packed than in control tissue. In the last stage (stage 4), large myelin sheaths are left with few wrappings before altogether disappearing. Note that it is not possible to follow the fate of individual axons but this pathological process is deduced from the appearance of the majority of axons after the injury. Scale bar is 10 µm for all micrographs.
(TIF)
