# Supplementary figures and images for: Pathological Changes in the White Matter after Spinal Contusion Injury in the Rat
Source: PLoS One. 2012 Aug 29;7(8):e43484. doi: 10.1371/journal.pone.0043484 (PMC3430695; doi:10.1371/journal.pone.0043484)

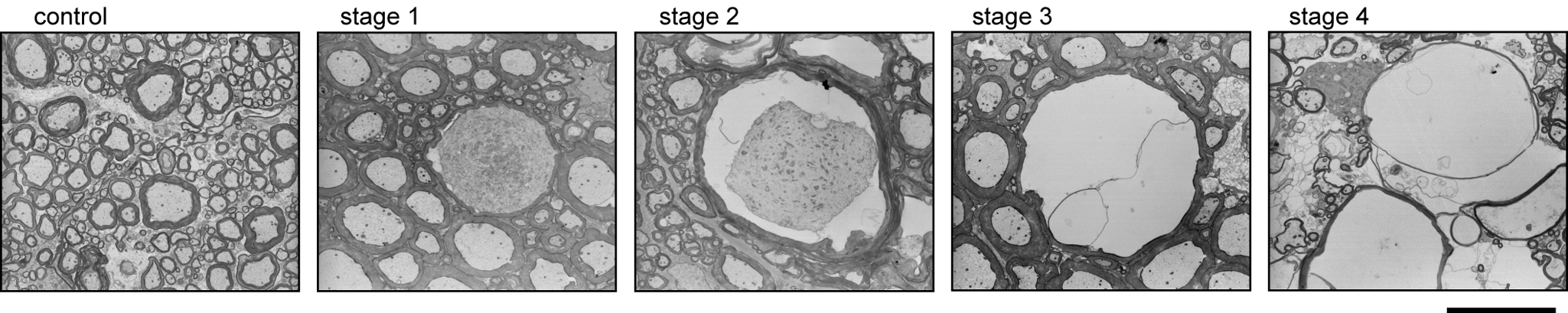

Supplement: Figure S1 — Area of myelinated axons in the dorsal column (A) and ventrolateral tracts (B) in control and spinal injured rats. In both dorsal column (DC) and ventrolateral tract (VLT) there is a trend for the area to be progressively smaller with time in the middle of the injury. At 9 mm rostral to the injury the area is similar to 0 week controls in all injury groups whereas at 9 mm caudal in the later injury groups (4 and 10 weeks) the area is closer to 10-week controls. Note that in 10-week controls the area is 67% more than in 0-week controls in the DC and 57% more in the VLT. (TIF) [file pone.0043484.s001.tif]

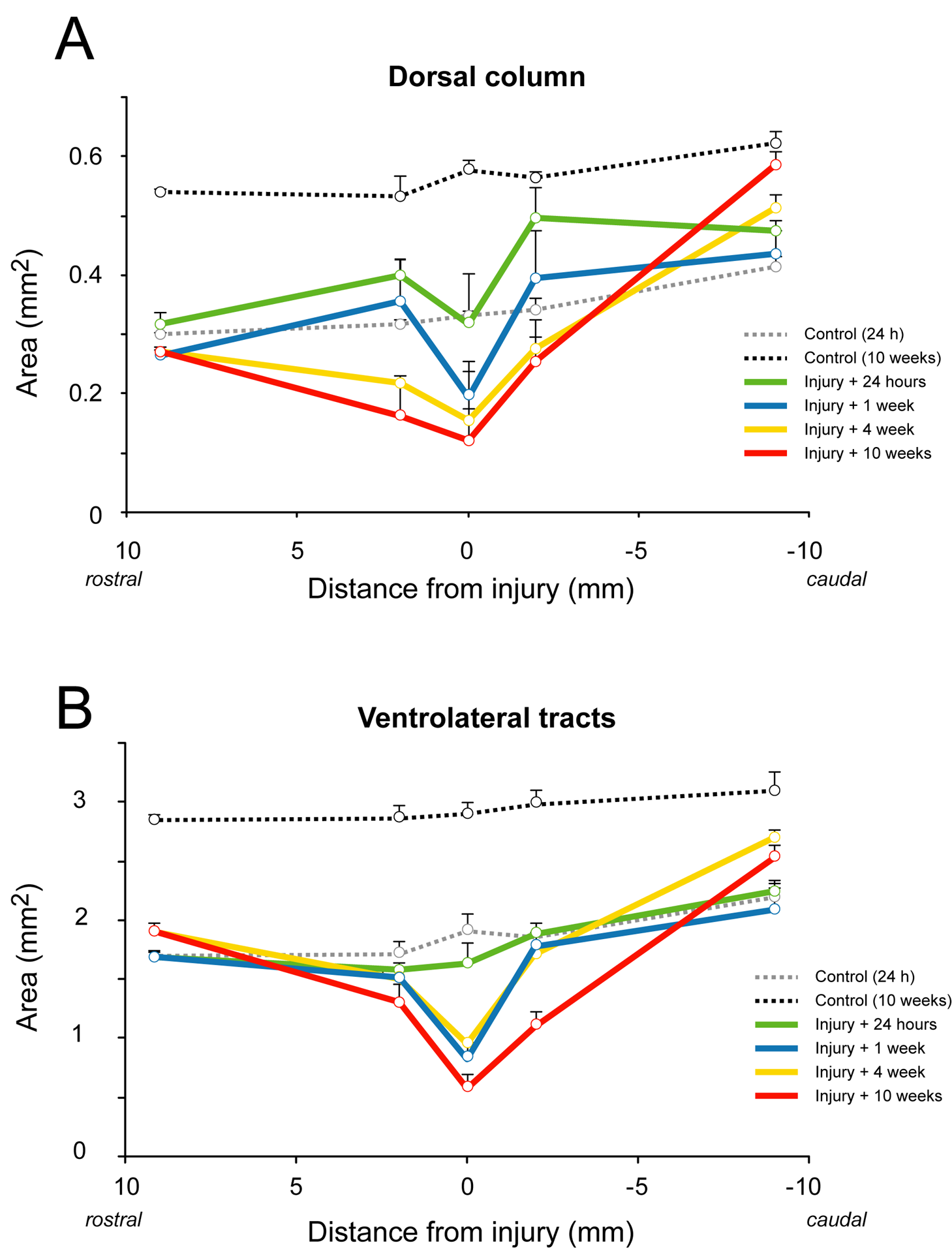

Supplement: Figure S2 — Electron micrographs of axons at different pathological stages, illustrating what appears to be a common pathological process of necrotic axons. In the control spinal cord the largest axons found within white matter are around 6–7 µm. After injury, many axons swell (stage 1) and the axoplasm becomes denser with organelles clustering in the centre of the axon. These swollen axons detach from the myelin sheath (stage 2) and the axoplasm becomes less dense compared to stage 1. The axon disintegrates and a microcyst is left in the space where the axons were situated (stage 3). It is interesting to note that morphologically intact axons are often found next to necrotic axons. The myelin sheath sometimes appears morphologically intact but wrappings are often less densely packed than in control tissue. In the last stage (stage 4), large myelin sheaths are left with few wrappings before altogether disappearing. Note that it is not possible to follow the fate of individual axons but this pathological process is deduced from the appearance of the majority of axons after the injury. Scale bar is 10 µm for all micrographs. (TIF) [file pone.0043484.s002.tif]
